# Melkersson-Rosenthal syndrome with facial swelling and palsy and associated diagnostic challenges: A case report

**DOI:** 10.3892/mi.2025.294

**Published:** 2025-12-31

**Authors:** Alina Teresa Sánchez Vázquez, Anahí Parcero Tamay, María J. Calvo Domínguez, Martha Patricia Pacheco Arenas, Valeria Iuxely Medrano Pichardo

**Affiliations:** 1 Department of Internal Medicine, ‘Dr. Belisario Domínguez’ General Hospital ISSSTE, Tuxtla Gutiérrez, Chiapas 29040, Mexico; 2 Department of Pediatrics, High Specialty Regional Hospital of Zumpango, Zumpango de Ocampo 55600, Mexico; 3 Department of Medicine, Clínica Hospital ISSSTE Río Bravo, Ciudad Río Bravo, Tamaulipas 88970, Mexico

**Keywords:** Melkersson-Rosenthal syndrome, facial paralysis, orofacial edema, granulomatous cheilitis, immunosuppressive therapy

## Abstract

Melkersson-Rosenthal syndrome (MRS) is a rare neuro-mucocutaneous disorder characterized by recurrent orofacial edema, peripheral facial palsy and a fissured tongue, although the complete triad is rarely observed. The present study reports the case of a 22-year-old male patient who initially presented with facial paralysis, later developing cheek edema, dermatosis and systemic symptoms; the histopathological findings consistent with granulomatous cheilitis. Laboratory analyses revealed a low C1q level and positive antinuclear antibody. Based on clinical and biopsy findings, MRS was diagnosed. The patient responded well to a 6-month regimen of deflazacort, epinastine and methotrexate. On the whole, the present case report illustrates an incomplete, yet clinically significant form of MRS. Diagnosis is primarily clinical, supported by histology and exclusion of similar conditions. Treatment focuses on symptom control with corticosteroids; immunomodulators or biologics may be used in refractory cases. Early recognition is essential, even in the absence of the full triad.

## Introduction

Melkersson-Rosenthal syndrome (MRS) is a rare neuro-mucocutaneous disorder characterized by a clinical triad consisting of recurrent orofacial edema, peripheral facial paralysis and a fissured tongue (1). However, the complete triad is observed in only 8 to 25% of cases, with monosymptomatic presentations being more common. Among these, orofacial edema is the predominant manifestation, occurring in ~80% of patients (2). The estimated prevalence of MRS is <0.1% in the general population. It is most frequently diagnosed in the second and third decades of life, and its occurrence during childhood or in older adults is exceptional (3). Pathophysiology remains poorly understood, although proposed mechanisms include an autosomal dominant inheritance with variable penetrance, along with possible contributions from immunologic, infectious, or allergic factors (4).

Diagnosis is primarily clinical and relies on identifying the characteristic signs, while excluding other causes of recurrent facial edema and facial palsy. In cases with atypical or incomplete presentations, the biopsy of affected tissues may be useful, often revealing a non-caseating granulomatous inflammatory infiltrate consistent with granulomatous cheilitis. However, these findings are not pathognomonic, and their absence does not rule out the diagnosis (5).

The management of MRS focuses on symptom control. Systemic or intralesional corticosteroids are the mainstay for treating acute episodes, providing favorable outcomes in reducing edema and facial palsy. In chronic or refractory cases, immunosuppressive agents such as methotrexate, azathioprine, or even biological therapies targeting tumor necrosis factor (anti-TNF) have been proposed. Surgical intervention may also be considered for patients with persistent lip edema or severe facial dysfunction unresponsive to medical therapy (6).

Given the broad clinical spectrum of MRS and the fact that the majority of patients do not present with the complete triad of symptoms, reporting atypical or incomplete cases is crucial to improving clinical recognition. These cases highlight the diagnostic challenges posed by overlapping symptoms with infectious, allergic, or autoimmune conditions and underscore the importance of a thorough differential diagnosis.

In this context, the present study reports the case of a 22-year-old male patient with facial swelling and palsy, whose diagnosis of MRS was particularly challenging due to the presence of systemic symptoms, such as fever and the absence of the classic fissured tongue. The present case report aimed to emphasize the atypical nature of the presentation, discuss the diagnostic difficulties, and provide educational value for clinicians who may encounter similar scenarios.

## Case report

**Figure 1:**
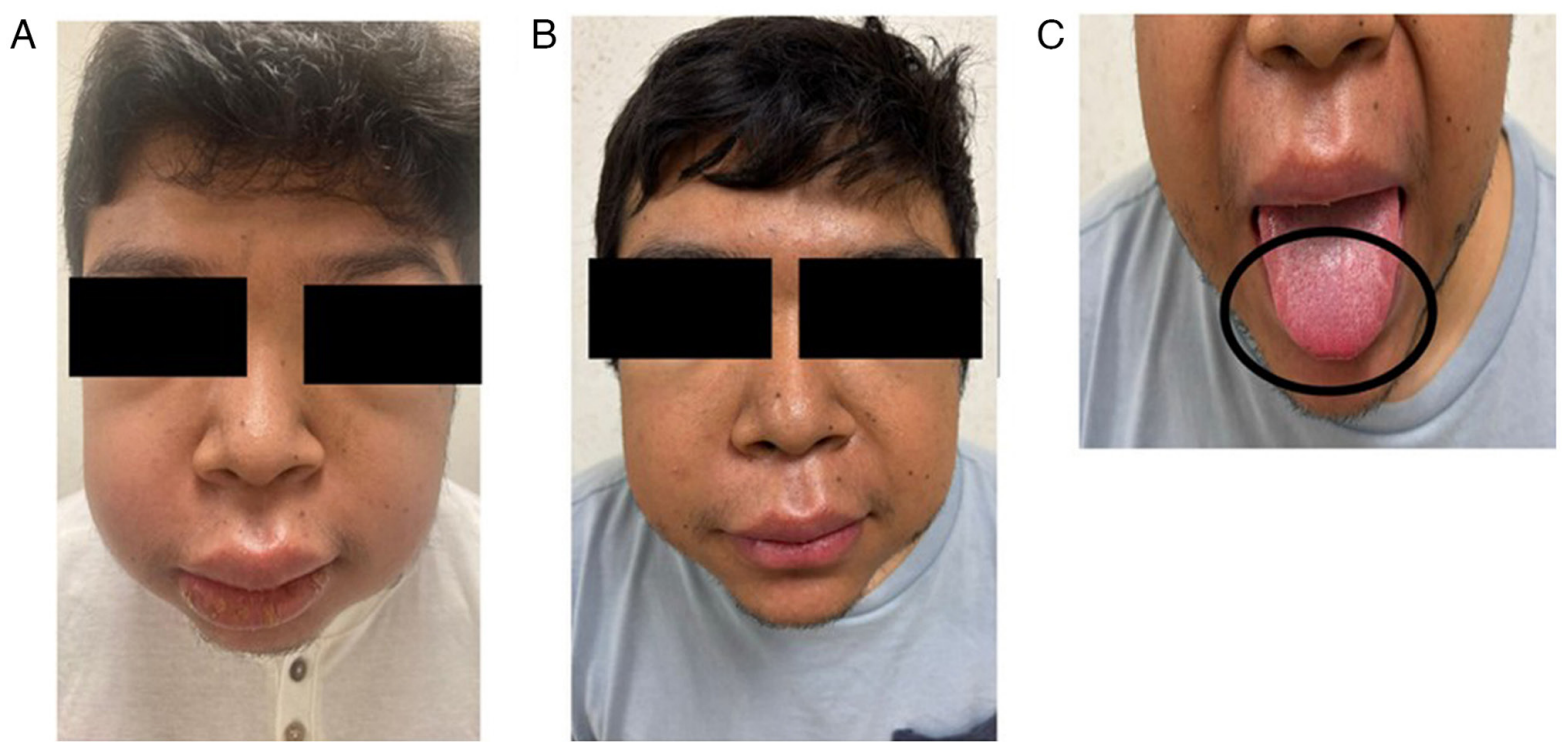
Clinical images illustrating the presentation and progression in a patient with Melkersson-Rosenthal syndrome. (A) Facial dermatosis characterized by localized facial edema at initial consultation. (B) Clinical improvement with visible reduction of edema after six months of treatment with methotrexate. (C) Presence of fissures on the lateral borders of the tongue (black circle).

The present study describes the case of a 22-year-old male patient from Tuxtla Gutiérrez, Chiapas, Mexico. The patient was a university student with no relevant medical history, who experienced a first episode of left-sided facial paralysis in June, 2022. He was treated at 'Dr. Belisario Domínguez’ General Hospital ISSSTE, Tuxtla Gutiérrez, Chiapas, Mexico with prednisone (5 mg) and antihistamines, exhibiting a favorable response. In November, 2022, he developed dermatosis characterized by edema of the right cheek, which progressively extended to the remainder of his face (Fig. 1). This was accompanied by intermittent fever reaching up to 38˚C, predominantly in the late afternoon and evening, which was resolved with antipyretics. The patient also reported unintentional weight loss and alopecia.

The diagnostic evaluation began in March, 2023. Laboratory tests revealed decreased C1q complement levels (54.3 mg/dl) and positive antinuclear antibody (ANA) with a titer of 1:80 and a cytoplasmic reticular immunofluorescence pattern. Anti-double-stranded DNA, anti-Ro, anti-La and ANCA antibodies were negative. In December, 2023, a biopsy of the lower lip mucosa was performed. A histological evaluation was performed on formalin-fixed, paraffin-embedded tissue sections of the lip mucosa. The samples were sectioned at a thickness of 4 µm, mounted on positively charged glass slides (Superfrost™ Plus, Thermo Fisher Scientific, Inc.) and dried overnight at 37˚C. The sections were then deparaffinized in xylene (cat. no. 534056, MilliporeSigma) and rehydrated through a graded ethanol series (100, 95 and 70%) to distilled water. Routine hematoxylin and eosin (H&E) staining was performed using Mayer's hematoxylin (cat. no. 1.09249.0500, Merck KGaA) and eosin Y alcoholic solution (cat. no. E4382, MilliporeSigma). The slides were subsequently dehydrated in ascending ethanol concentrations, cleared in xylene and mounted with synthetic resin (cat. no. 1.07961.0100, Entellan™ New, Merck KGaA).

**Figure 2:**
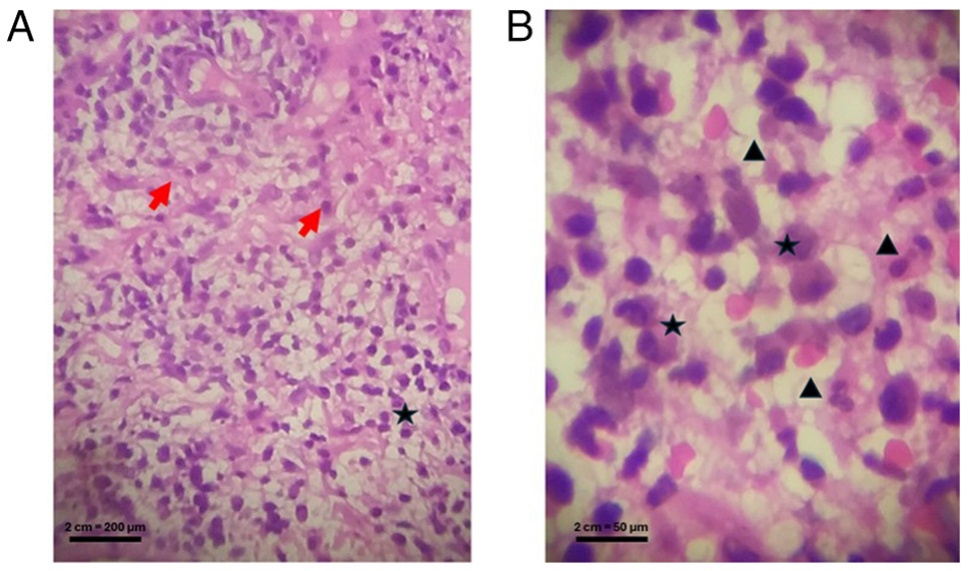
Histopathological findings of lower lip mucosa biopsy in Melkersson-Rosenthal syndrome. (A) Moderate irregular acanthosis (red arrows) and focal hydropic degeneration of the basal layer (black star) (magnification, x10). (B) Inflammatory infiltrate composed of lymphocytes, histiocytes, neutrophils and plasma cells, focal hydropic degeneration of the basal layer (black star) and mixed inflammatory infiltrate (black arrowhead) and dilated, congested blood vessels (magnification, x40).

No immunohistochemical staining was applied to this specimen. Histopathological analysis revealed moderate irregular acanthosis, focal hydropic degeneration of the basal layer, and a dense mixed inflammatory infiltrate composed of lymphocytes, histiocytes, neutrophils and plasma cells, along with dilated and congested blood vessels (Fig. 2).

Based on the clinical presentation and histopathological findings, a diagnosis of MRS was established. Treatment was initiated in January, 2024 with deflazacort at 12 mg/day, epinastine at 20 mg/day and methotrexate at 10 mg/week for 6 months, resulting in a marked clinical improvement of the dermatosis without any side-effects (Fig. 1B).

## Discussion

The present study describes the case of a young male patient with a history of unilateral facial paralysis who subsequently developed persistent facial edema; the histopathological findings consistent with granulomatous cheilitis, and favorable clinical improvement following treatment with corticosteroids and immunomodulators. These elements led to the diagnosis of MRS, a rare condition whose incomplete presentation may hinder timely recognition.

MRS is an uncommon neuro-mucocutaneous disorder characterized by the classic triad of recurrent orofacial edema, peripheral facial palsy and fissured tongue. Although it can occur at any age, it is most prevalent between the second and fourth decades of life, with a 2:1 female predominance (X-5). Pediatric cases are rare, but have been reported in children between 7 and 12 years of age. The overall incidence in the general population is estimated to be approximately 0.08% (7).

The etiology of MRS remains unclear, although several predisposing factors have been proposed. Infectious agents, such as Epstein-Barr virus, cytomegalovirus and SARS-CoV-2 have been implicated, along with bacteria such as *Mycobacterium tuberculosis*. Associations have also been described with atopy, food allergies, exposure to environmental antigens and hypersensitivity reactions to monosodium glutamate; however, conclusive evidence of causality is lacking (8,9). Dysregulated immune responses are also considered relevant (10,11).

Clinically, MRS may present in complete or partial forms. The classic triad of symptoms is present in only 8 to 18% of cases. The most common manifestation is recurrent orofacial edema, typically painless, unilateral, and most frequently affecting the upper lip. Edema may extend to the cheeks, palate, tongue, gums, periorbital region, pharynx, or larynx (12). Facial palsy occurs in 50 to 90% of cases and is generally unilateral and transient, while a fissured tongue is observed in up to 40% of patients (13-15).

The diagnosis of MRS poses a clinical challenge due to the absence of standardized diagnostic criteria or specific biomarkers. Diagnosis is primarily clinical, based on the recognition of characteristic signs and exclusion of other conditions with similar symptoms. Biopsy may be useful in atypical or incomplete cases. In the early stages, histological findings are often non-specific, featuring edema and a mixed inflammatory infiltrate. In advanced stages, non-caseating granulomas may be observed, such as those observed in sarcoidosis or Crohn's disease (16).

In the patient described herein, the presence of fever and the absence of fissured tongue complicated the diagnostic process, as systemic symptoms such as fever are rarely reported in MRS. This required a careful differential diagnosis, including infectious etiologies, such as bacterial abscesses, viral reactivations and Hansen's disease, as well as non-infectious causes such as sarcoidosis, angioedema, Crohn's disease and lymphoproliferative disorders. Ultimately, the combination of persistent orofacial edema, recurrent facial palsy and histopathological features consistent with granulomatous cheilitis supported the diagnosis of MRS.

The differential diagnosis is broad and should include hereditary or acquired angioedema, Hansen's disease, sarcoidosis, dental absences, lymphomas, trauma and other granulomatous disorders, such as Wegener's granulomatosis, amyloidosis, specific infections, or foreign body reactions (17). There is no standardized treatment for MRS; management focuses on symptom control. Systemic or intralesional corticosteroids remain the cornerstone of therapy, with recommended courses lasting 3 to 6 weeks using prednisone or triamcinolone in combination with antihistamines (18). Antibiotics may be indicated in cases with bacterial superinfection. Immunomodulatory agents such as azathioprine, methotrexate, thalidomide, tacrolimus and antimalarials such as chloroquine have exhibited efficacy in refractory cases (19). Biological therapies such as infliximab or adalimumab (anti-TNF-α) have also demonstrated benefit. Physical therapy may help preserve muscular function in patients with facial paralysis. In most cases, facial palsy is resolved spontaneously within approximately three weeks. Reconstructive surgery is reserved for cases with persistent or refractory edema, while surgical decompression of the facial nerve may be considered in selected cases when compression is presumed to be the underlying mechanism (12).

In the patient in the present study, corticosteroid therapy led to clinical improvement; however, methotrexate was introduced as a steroid-sparing agent to minimize the risks of long-term corticosteroid exposure. Although methotrexate is not universally required in MRS, its use has been supported in recurrent or persistent cases, and previous reports describe its efficacy as part of immunomodulatory therapy (20-22). This rationale guided the therapeutic decision for the present case.

Compared with previous case reports, the novelty of the present case report lies in the atypical presentation, particularly the association with systemic symptoms (fever), an incomplete clinical triad and inconclusive early histopathology. By highlighting these features, the present case report adds educational value for clinicians and underscores the need to consider MRS even in patients who do not fully meet the classic diagnostic triad (23).

The present case report has several limitations which should be mentioned. First, the absence of long-term follow-up data precludes evaluation of recurrence patterns, sustained treatment response, and potential late complications. Second, as with all single-patient case reports, the findings cannot be generalized, and causality between clinical manifestations and therapeutic outcomes cannot be firmly established. Finally, histopathological findings in this case were not pathognomonic, which reflects the inherent diagnostic uncertainty in atypical forms of MRS. Despite these limitations, the present case report underscores the importance of documenting incomplete or unusual presentations of MRS, particularly those associated with systemic symptoms such as fever, which are rarely described in the literature. From a clinical perspective, the present case report highlights the need for heightened awareness of diagnostic challenges and for careful differential diagnosis in patients presenting with orofacial edema and facial palsy (24,25). Future research is required to focus on larger case series or prospective registries to better characterize the full clinical spectrum of MRS, identify reliable diagnostic biomarkers, and evaluate the long-term efficacy and safety of different therapeutic strategies, including corticosteroid-sparing immunomodulators such as methotrexate.

In conclusion, the present case report highlights the importance of including MRS in the differential diagnosis of recurrent facial paralysis and persistent orofacial edema, even in the absence of the complete triad of symptoms. A histopathological evaluation and a comprehensive clinical approach are essential for timely diagnosis and appropriate management. Documenting atypical cases such as the one presented herein contributes to a broader understanding of this rare condition and may facilitate its recognition in unconventional clinical scenarios.

## Data Availability

The data generated in the present study may be requested from the corresponding author.
